# 3DWaFusion: Three-Dimensional Multiscale Wavelet Convolutional Neural Network for Multimodal Medical Image Fusion

**DOI:** 10.3390/s26123784

**Published:** 2026-06-14

**Authors:** Yu Wang, Rui Zhang, Zhiqiang Zhang, Ningzhong Liu, Xiulai Wang

**Affiliations:** 1 MIIT Key Laboratory of Pattern Analysis and Machine Intelligence, College of Computer Science and Technology, Nanjing University of Aeronautics and Astronautics, Nanjing 211106, China; wangyucs@nuaa.edu.cn (Y.W.); zhangruics@nuaa.edu.cn (R.Z.);; 2 Jinling Clinical Medical College, Nanjing University of Aeronautics and Astronautics, Nanjing 211106, China

**Keywords:** multimodal image fusion, 3D wavelet network, global and local feature calibration

## Abstract

Multimodal image fusion is a promising technology designed to fuse information from different medical sensors, which offer structured insights for disease diagnosis and treatment. However, existing 2D-centric fusion methods fail to capture 3D spatial continuity, and conventional wavelet-based approaches lack adaptability to diverse lesion regions and suffer from background artifacts. To address this issue, we propose a 3D multiscale wavelet convolutional neural network for multimodal medical image fusion. Specifically, a 3D Discrete Wavelet Transformation (3D DWT) is introduced to decompose input volumes into multi-frequency bands, isolating anatomical structures and lesion details while reducing 3D spatial redundancy. We embed hierarchical multiple frequency band into a Global and Local Feature Calibration (GLFC) module to adaptively enhance single-modal features by fusing global contextual information and local details. Furthermore, a pyramid group-wise multiscale feature interaction is proposed for capturing complementary features across different spatial scales. Finally, a voxel-wise weighted averaging strategy reconstructs the fused image by adaptively assigning contributions to each modality at every spatial position, effectively eliminating artifacts and improving the visual fidelity of the result. Extensive experiments on the BraTS2020 and Hecktor datasets demonstrate that our proposed method outperforms state-of-the-art (SOTA) fusion methods in both subjective visual quality and objective metrics. Moreover, downstream segmentation validation confirms that fused images from our method significantly improve tumor segmentation accuracy. The source code and pre-trained models will be publicly available.

## 1. Introduction

Driven by the potent capabilities of Deep Learning (DL) [1], multimodal medical image fusion has emerged as a pivotal research frontier in medical image analysis [2,3]. Clinical mainstream 3D imaging modalities include Magnetic Resonance Imaging (MRI), functional MRI (fMRI), Computed Tomography (CT), and Positron Emission Tomography (PET) [4,5]. These modalities provide highly complementary information including anatomical structures, soft-tissue characteristics, neurofunctional activity, and metabolic molecular levels. Specifically, MRI imaging technology offers superior soft-tissue contrast and functional insights. CT imaging technology provides high-resolution visualization of dense anatomical structures. fMRI imaging technology captures neurovascular coupling and brain activity, while PET imaging technology reflects metabolic and physiological processes but lacks precise anatomical localization [6]. With the continuous evolution of medical sensors, single-modality imaging often fails to simultaneously capture multidimensional information. Multimodal image fusion addresses this limitation by integrating complementary feature from different medical sensors to improve diagnostic accuracy and support more precise downstream applications [7,8].

Currently, most research and clinical applications focus on 2D medical image fusion [9,10]. In contrast, 3D multimodal medical image fusion [11] enables more accurate anatomical representation and richer spatial context, aligning better with clinical requirements in diagnosis. Therefore, developing robust 3D multimodal fusion methods that effectively exploit spatial information is of significant importance, and it provides clinical value for advancing precision radiotherapy and intelligent decision support systems.

Over the past decade, the multimodal medical image fusion has evolved from traditional optimization based to Deep Learning (DL). With empowering Convolutional Neural Networks (CNNs), some works combined Siamese CNNs with contrast pyramid decomposition successfully achieved pixel-level weight learning to preserve structural integrity [12]. The others integrated the Non-Subsampled Contourlet Transform (NSCT) with high-frequency-aware CNNs to adaptively enhance edge and texture representation in the transform domain [13]. Based on decomposition methodology, Zhao et al. [14] proposed a novel Correlation-Driven feature Decomposition Fusion (CDDFuse) network that explicitly disentangles multimodal features into modality-shared structural components and modality-specific detail components. Xu et al. [10] proposed a unified unsupervised image fusion network that adopts an adaptive feature fusion strategy to handle multimodal image fusion tasks with a single model without task-specific training.

To overcome the inherent limitations of CNNs in capturing long-range dependencies, hybrid architectures integrating CNNs and Transformers [15,16,17,18] have been proposed by employing different heuristic design or attention mechanism. More recently, State Space Models (SSMs) [19] have been introduced for demonstrating superior potential over traditional CNNs and Transformers in multi-contrast MRI and MRI–CT fusion tasks. Generative Adversarial Networks (GANs) [20] and a diffusion model [21] with generative capabilities have proven effective in improving anatomical-functional representation and low-dose imaging enhancement. While these approaches effectively capture slice-level features, they fail to model 3D spatial relationships. It is inevitable that these 2D medical image fusion would lead to inter-slice information loss and spatial inconsistency when applied to 3D volumetric data.

To bridge this gap, research is aggressively transitioning toward 3D multimodal medical image fusion. While early 3D CNNs and recent 3D-Mamba variants have attempted to enhance spatial context through voxel-level modeling. Liu et al. [22] proposed an end-to-end multimodal MRI volumetric data fusion network with an attention-based multimodal feature fusion module and modality-aware loss, explicitly designed to maintain 3D anatomical structure. Liu et al. [22] combined CNN and Mamba structures to improve anatomical continuity. Zou et al. [23] proposed MMR-Mamba that leverages the Mamba architecture with spatial-frequency information fusion to achieve superior reconstruction quality. Liu et al. [24] proposed MambaDiff, which integrates a Mamba-enhanced architecture with diffusion models through a semantic hierarchical embedding mechanism and global-slice perception to improve structural consistency in 3D medical image.

However, current 3D multimodal medical image fusion methods, typically based on volumetric CNNs or hybrid CNN–Transformer architectures, still operate directly in the voxel domain and must process highly redundant 3D feature maps, which leads to heavy computation burdens and limited redundancy suppression in high-dimensional volumes. While the Discrete Wavelet Transform (DWT) has been widely exploited in 2D multimodal fusion and segmentation [25,26], its extension to volumetric medical fusion remains largely under-explored. This is similar to [25], where DWT was integrated into each scale of a multi-scale encoder–decoder to decompose features into high- and low-frequency subbands and then frequency-aware fusion in 2D medical image fusion was performed. However, existing wavelet-based fusion and segmentation networks mainly focus on planar images and limit wavelet usage to simple downsampling or band-wise processing [27]. Even in related 3D tasks, recent architectures like MambaDiff [24] have focused on global-slice perception via State Space Models, yet they do not explicitly address high-dimensional data redundancy at a multi-scale level.

**Figure 1:**
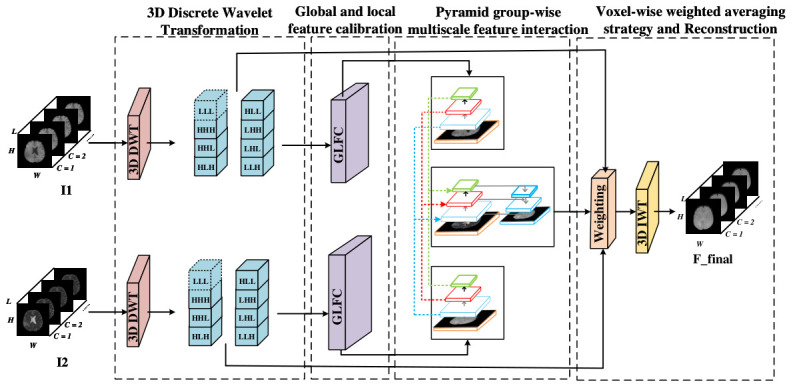
Overview of the proposed 3DWaFusion framework. It consists of four core stages: 3D discrete wavelet transformation (3D DWT) for multi-frequency decomposition, global and local feature calibration (GLFC) for single-modal enhancement, pyramid group-wise multiscale feature interaction (PGMF) for cross-modal information fusion, and voxel-wise weighted averaging with 3D inverse DWT (3D IDWT) for artifact-free reconstruction.

Despite the progress of multimodal medical image fusion, existing methods still face three critical limitations: (1) 2D-centric fusion paradigm fails to capture 3D spatial continuity, leading to discontinuity anatomical structures in fused volumes. (2) Conventional wavelet-based methods rely on fixed fusion rules, lacking adaptability to diverse lesion regions and imaging modalities. Addressing these gaps, this work proposes a 3D Multi-scale Wavelet Convolutional Neural Network (3DWaFusion) shown in Figure 1.

Overall, the main contributions can be summarized as follows:

- A novel 3D multi-scale wavelet convolutional neural network (3DWaFusion) is proposed for multimodal medical image fusion to achieve efficient redundant information suppression and salient feature enhancement.
- The designed GLFC module with dual parallel calibration branches effectively solves the problems of inconsistent feature distribution and unbalanced global-local emphasis, providing high-quality calibrated features for subsequent multimodal interaction.
- The PGMF module realizes targeted multi-scale and cross-modal feature interaction through frequency band grouping and pyramid construction, significantly improving the complementarity and discriminability of multimodal fusion features.

The remainder sections of this paper are organized as follows. Section 2 describes the proposed 3DWaFusion framework in detail. Section 3 presents the experimental setup, evaluation metrics, and comparative results against state-of-the-art approaches, followed by ablation studies and qualitative analyses. Finally, Section 4 concludes this paper and discusses future work.

## 2. The Proposed 3DWaFusion

### 2.1. The Overview of Our Proposed Method

Given 3D multimodal medical images $I_{1},I_{2}\in R^{1\times L\times H\times W}$ as input, we propose a 3D multiscale wavelet convolutional neural network (3DWaFusion) for multimodal medical image fusion. As shown in Figure 1, the proposed 3DWaFusion follows a sequential pipeline consisting of the following major stages: 3D DWT-based wavelet decomposition, GLFC-based global-local feature calibration, PGMF-based pyramid group-wise multiscale interaction, voxel-wise weighted fusion, and 3D IDWT reconstruction for fusion. The proposed 3DWaFusion framework is trained in an end-to-end manner. Specifically, the 3D DWT sequentially performs 1D wavelet decomposition along the width, height, and depth dimensions of 3D medical volumes, effectively decoupling global structural information and local detailed features while reducing information redundancy. The GLFC module first splits the wavelet-decomposed features via depthwise separable convolution (DSC), then employs dual parallel branches to calibrate feature distributions, enhancing feature discriminability and consistency. Furthermore, the PGMF module conducts frequency band grouping and multi-scale pyramid construction, followed by intra-group cross-scale interaction and inter-group cross-modal interaction, fully exploiting complementary information between different modalities and scales. Finally, the voxel-wise weighted averaging strategy fuses the interacted features, and 3D IDWT reconstructs the final fused volume, ensuring the preservation of anatomical structures and fine details.

First, 3D DWT sequentially decomposes each input volume $I_{i}$ (i=1,2) along three spatial dimensions to generate multi-frequency band features $I_{i}^{whl}\in R^{8C\times L/2\times H/2\times W/2}$, which effectively decouple global structural information and local details while reducing redundancy. Second, the GLFC module takes the decomposed features $I_{i}^{whl}$ as input and performs dual-branch global-local feature calibration, outputting enhanced single-modal features $F_{i}\in R^{C^{\prime }\times L/2\times H/2\times W/2}$ for each modality. Third, the PGMF module conducts group-wise multi-scale extraction and cross-modal interaction on the calibrated features $F_{1}$ and $F_{2}$, yielding a compact and highly representative fused feature $F_{\mathrm{PGMF}}$ with integrated multi-scale contextual information. The fused feature $F_{\mathrm{PGMF}}$ is fed into a lightweight weight generation branch to produce a voxel-wise weight mask $m\in {\left[0,1\right]}^{1\times L/2\times H/2\times W/2}$ that adaptively measures the contribution of each modality at every spatial position. The parameter m in Equation (1) is not a manually selected hyperparameter. Instead, it is an adaptive voxel-wise weight mask learned by the proposed network. The fused wavelet-domain feature is obtained through voxel-wise weighted averaging:

(1) $$I_{f}=m⊙I_{1}^{whl}+\left(1-m\right)⊙I_{2}^{whl}$$

where ⊙ denotes element-wise multiplication. The final fused volume $\hat{I}\in R^{1\times L\times H\times W}$ is reconstructed by 3D IDWT, which perfectly preserves zero-intensity background regions and eliminates spurious non-zero voxels.

### 2.2. Three-Dimensional Discrete Wavelet Transformation (3D DWT)

**Figure 2:**
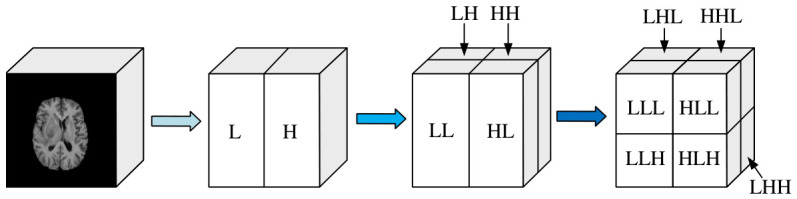
Illustration of the 3D Discrete Wavelet Transform (3D DWT) decomposition process. The input 3D medical volume is sequentially decomposed along three spatial dimensions (width, height, and depth), yielding one low-frequency subband (LLL) that encodes global anatomical structures and seven high-frequency subbands (HLL, LHL, LLH, HHL, HLH, LHH, HHH) that capture fine-grained local details (e.g., edges and textures) at different scales and orientations.

As the fundamental frequency-domain decomposition unit of our proposed 3D multiscale wavelet convolutional neural network (3DWaFusion), similar to [28], we apply 3D discrete wavelet transformation (3D DWT) that is devised to excavate multi-scale spatial structures and eliminate redundant information in 3D multimodal medical volumes. This process effectively separates low-frequency global structures and high-frequency details without introducing excessive redundancy. The details can be found in Figure 2. Given an input 3D medical image $I_{m}\in R^{1\times L\times H\times W}\;\left(m=1,2\right)$ of the *m*th modality, where *L*, *H*, *W* represent the depth, height and width of the volumetric data, respectively, and 3D DWT executes 1D discrete wavelet transform sequentially along depth, height and width dimensions to achieve hierarchical multi-frequency decomposition.

Specifically, 3D DWT decomposes the input volume into eight distinct frequency sub-bands, including one low-frequency sub-band (LLL) that encodes global anatomical structures and seven high-frequency sub-bands (HHH,HHL,HLH,HLL,LHH,LHL,LLH) that capture fine textures, edges and local detail variations. After decomposition, the spatial resolution of each frequency sub-band is reduced to 1/2 of the input, i.e., $R^{1\times L/2\times H/2\times W/2}$, which effectively reduces computational burden while retaining complete contextual information. The multi-frequency sub-band set generated by 3D DWT for the *m*th modality is formally defined as:

(2) $$B_{m}=\left\{B_{m,\mathrm{LLL}},B_{m,\mathrm{HHH}},B_{m,\mathrm{HHL}},B_{m,\mathrm{HLH}},B_{m,\mathrm{HLL}},B_{m,\mathrm{LHH}},B_{m,\mathrm{LHL}},B_{m,\mathrm{LLH}}\right\}$$

where $B_{m,\cdot }$ denotes the frequency sub-band feature of the corresponding component.

Mathematically, the 3D DWT first operates on the width dimension (*W*) of $I_{i}$ using 1D DWT, denoted as $DWT_{1d}\left(\cdot ,\cdot \right)$. Here, $DWT_{1d}\left(input,dim\right)$ performs 1D wavelet decomposition along the specified dimension dim. The output of this step is $I_{i}^{w}$ formulated as:

(3) $$I_{i}^{w}=DWT_{1d}\left(I_{i}\right)$$

where $I_{i}^{w}\in R^{2\times C\times L\times H\times \frac{W}{2}}$ contains the low-frequency and high-frequency coefficients after decomposition along the width dimension. We then concatenate the first two dimensions (i.e., the decomposed coefficient dimension and the original channel dimension) using the flatten(1,2) operation, resulting in a feature tensor of size $R^{2C\times L\times H\times \frac{W}{2}}$.

Next, we apply 1D DWT on the height dimension (*H*) of $I_{i}^{w}$ to obtain the intermediate feature:

(4) $$I_{i}^{w\to h}={\mathrm{DWT}}_{1d}\left(I_{i}^{w}\right)$$

Subsequently, we flatten and concatenate the first two dimensions of $I_{i}^{w\to h}$ to unify the channel representation:

(5) $$I_{i}^{wh}=\mathrm{flatten}\left(I_{i}^{w\to h}\right)$$

yielding a tensor with shape $R^{4C\times L\times \frac{H}{2}\times \frac{W}{2}}$. One-dimnsional DWT is performed on the depth dimension (*L*) of $I_{i}^{wh}$:

(6) $$I_{i}^{wh\to l}={\mathrm{DWT}}_{1d}\left(I_{i}^{wh}\right)$$

Subsequently, we adopt flatten(1,2) to concatenate the first two dimensions and derive the final decomposed feature $I_{i}^{whl}$:

(7) $$I_{i}^{whl}=\mathrm{flatten}\left(I_{i}^{wh\to l}\right)$$

After dimension fusion, the output feature tensor has a size of $R^{8C\times \frac{L}{2}\times \frac{H}{2}\times \frac{W}{2}}$, which corresponds to the eight distinct frequency sub-bands, including one low-frequency LLL component and seven high-frequency components.

By decoupling structural and detailed information in the frequency domain through sequential 1D DWT operations, the 3D DWT provides compact and discriminative multi-frequency feature inputs for the subsequent Global and Local Feature Calibration (GLFC) module. The calibrated features are then delivered to the Pyramid Group-wise Multiscale Feature Interaction (PGMF) module for multimodal and multi-scale feature fusion, laying a solid foundation for the overall multimodal medical image fusion framework of 3DWaFusion.

### 2.3. Global and Local Feature Calibration (GLFC)

**Figure 3:**
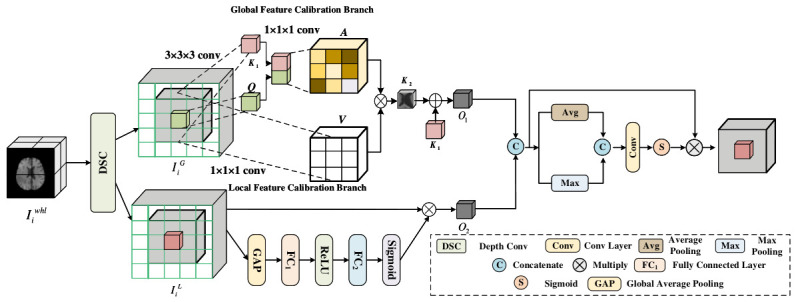
Illustration of the proposed Global and Local Feature Calibration (GLFC) module. Taking a multi-frequency band feature $I_{i}^{\mathrm{whl}}$ (output from the 3D DWT decomposition) as input, the module first splits the feature into two parallel branches via depthwise separable convolution (DSC): the Global Feature Calibration Branch and the Local Feature Calibration Branch. The outputs $O_{1}$ and $O_{2}$ are concatenated, then refined by a spatial attention module combining average and max pooling operations to generate the final calibrated feature, effectively integrating global contextual information and local structural details for subsequent multimodal feature interaction.

Following the 3D DWT decomposition, the multi-band frequency feature $I_{i}^{whl}$ with rich anatomical structures and textural details but lacks adaptive calibration between global contextual dependencies and local fine-grained details. To address this issue, we propose the Global and Local Feature Calibration (GLFC) module shown in Figure 3, which adopts dual parallel branches to perform feature calibration and enhancement for each modality, providing compact features for the subsequent PGMF module.

Formally, the input of GLFC is the 3D DWT output of the *i*th modality: $I_{i}^{whl}\in R^{8C\times L/2\times H/2\times W/2}$ (i=1,2), where *i* denotes the modality index, 8C is the concatenated channel dimension of eight frequency bands, and L/2×H/2×W/2 is the unified spatial resolution after wavelet decomposition. First, a depthwise separable convolution (DSC) is applied to split $I_{i}^{whl}$ into two parallel branch inputs:

(8) $$I_{i}^{G},I_{i}^{L}=\mathrm{DSC}\left(I_{i}^{whl}\right)$$

where $I_{i}^{G}$ is fed into the global feature calibration branch and $I_{i}^{L}$ into the local feature calibration branch.

#### 2.3.1. Global Feature Calibration Branch

This branch models long-range contextual dependencies via a 3D self-attention mechanism to capture global anatomical structures. First, $I_{i}^{G}$ is processed by 3×3×3 and 1×1×1 convolutions to generate query (*Q*), key ($K_{1}$), and value (*V*) features:

(9) $$Q={\mathrm{Conv}}_{3\times 3\times 3}^{Q}\left(I_{i}^{G}\right)$$

(10) $$K_{1}={\mathrm{Conv}}_{3\times 3\times 3}^{K}\left(I_{i}^{G}\right)$$

(11) $$V={\mathrm{Conv}}_{1\times 1\times 1}^{V}\left(I_{i}^{G}\right)$$

The attention map *A* is computed by matrix multiplication and softmax normalization:

(12) $$A=\mathrm{Softmax}\left(\frac{Q^{⊤}K_{1}}{\sqrt{d}}\right)$$

where *d* is the dimension of *Q* and $K_{1}$. The attention-weighted feature is then obtained by multiplying *A* with *V*, followed by a residual connection with $K_{1}$ and a 3×3×3 convolution to produce the global-calibrated feature $O_{1}$:

(13) $$O_{1}={\mathrm{Conv}}_{3\times 3\times 3}\left(A⊙V+K_{1}\right)$$

#### 2.3.2. Local Feature Calibration Branch

This branch refines local spatial details (edges, textures) using channel-wise attention. First, 3D global average pooling (GAP) compresses $I_{i}^{L}$ into a compact channel descriptor $G_{i}\in R^{C_{L}}$:

(14) $$G_{i}=\mathrm{GAP}\left(I_{i}^{L}\right)$$

Two fully connected (FC) layers with ReLU and Sigmoid activations generate channel-wise attention weights:

(15) $$W_{i}=\sigma \left({\mathrm{FC}}_{2}\left(\delta \left({\mathrm{FC}}_{1}\left(G_{i}\right)\right)\right)\right)$$

where δ denotes ReLU activation and σ denotes Sigmoid activation. The local-calibrated feature $O_{2}$ is obtained by channel-wise multiplication between $W_{i}$ and $I_{i}^{L}$:

(16) $$O_{2}=W_{i}⊙I_{i}^{L}$$

where ⊙ represents channel-wise multiplication. The global-calibrated feature $O_{1}$ and local-calibrated feature $O_{2}$ are concatenated along the channel dimension:

(17) $$F_{\mathrm{cat}}=\left[O_{1};O_{2}\right]$$

where [·;·] denotes channel-wise concatenation. Subsequently, a spatial attention module is applied to refine $F_{\mathrm{cat}}$:

(18) $$F_{\mathrm{avg}}=\mathrm{AvgPool}(F_{\mathrm{cat}})$$

(19) $$F_{\max}=\mathrm{MaxPool}(F_{\mathrm{cat}})$$

(20) $$F_{\mathrm{spatial}}=\sigma \left({\mathrm{Conv}}_{1\times 1\times 1}\left(\left[F_{\mathrm{avg}};F_{\max}\right]\right)\right)$$

where AvgPool and MaxPool are average and max pooling operations, respectively. The final calibrated feature of the *i*th modality is obtained by element-wise multiplication between $F_{\mathrm{cat}}$ and $F_{\mathrm{spatial}}$:

(21) $$F_{i}=F_{\mathrm{cat}}⊙F_{\mathrm{spatial}}$$

where $F_{i}\in R^{C^{\prime }\times L/2\times H/2\times W/2}$ is the output of GLFC for the *i*th modality. After processing both modalities, the calibrated features $F_{1}$ and $F_{2}$ are fed into the subsequent Pyramid Group-wise Multiscale Feature Interaction (PGMF) module for multimodal and multi-scale feature fusion.

### 2.4. Pyramid Group-Wise Multiscale Feature Interaction (PGMF) Module

The calibrated features output by the GLFC module, $F_{1}$ and $F_{2}$, contain rich anatomical structures and local details for each modality but lack effective multi-scale and cross-modal interaction. To address this, inspired by the existing pyramid mechanism, we propose the Pyramid Group-wise Multiscale Feature Interaction (PGMF) module, which leverages a group-wise multi-scale extraction strategy, intra-group cross-modal interaction, and pyramid progressive fusion to achieve efficient multimodal feature integration.

To obtain group-wise multi-scale feature layers for each single modality, the input calibrated features $F_{1}$ and $F_{2}$ are first fed into three parallel 3D convolutional branches with kernel sizes of 1×1×1, 3×3×3, and 7×7×7, respectively. Each branch acts as an independent group to extract features with different receptive fields and resolutions, achieving group-wise multi-scale feature decoupling. The process is formulated as:

(22) $$F_{F_{1}}^{1}=\mathrm{Conv}1\left(F_{1}\right),\;F_{F_{2}}^{1}=\mathrm{Conv}1\left(F_{2}\right)$$

(23) $$F_{F_{1}}^{2}=\mathrm{Conv}3\left(F_{1}\right),\;F_{F_{2}}^{2}=\mathrm{Conv}3\left(F_{2}\right)$$

(24) $$F_{F_{1}}^{3}=\mathrm{Conv}7\left(F_{1}\right),\;F_{F_{2}}^{3}=\mathrm{Conv}7\left(F_{2}\right)$$

where Conv1, Conv3, and Conv7 denote 3D convolutions with kernel sizes of 1×1×1, 3×3×3, and 7×7×7, respectively.

Next, we perform *intra-group cross-modal interaction* to fuse features from different modalities within the same group, avoiding interference between different scale groups. Specifically, features from the same group of two modalities are concatenated along the channel dimension, and a 1×1×1 3D convolution is applied for channel compression and feature refinement:

(25) $$F_{F_{1},F_{2}}^{1}=\mathrm{Conv}1\left(\left[F_{F_{1}}^{1};F_{F_{2}}^{1}\right]\right)$$

(26) $$F_{F_{1},F_{2}}^{2}=\mathrm{Conv}1\left(\left[F_{F_{1}}^{2};F_{F_{2}}^{2}\right]\right)$$

(27) $$F_{F_{1},F_{2}}^{3}=\mathrm{Conv}1\left(\left[F_{F_{1}}^{3};F_{F_{2}}^{3}\right]\right)$$

where [·;·] denotes channel-wise concatenation.

Finally, a pyramid progressive fusion strategy is adopted to integrate multi-scale information. The deepest feature $F_{F_{1},F_{2}}^{3}$ is upsampled (UP) to restore the spatial resolution and added with $F_{F_{1},F_{2}}^{2}$ to obtain $F_{F_{1},F_{2}}^{2^{\prime }}$. After refinement by a 1×1×1 convolution, the same process is applied to fuse with $F_{F_{1},F_{2}}^{1}$, yielding the final multi-scale enriched feature $F_{\mathrm{PGMF}}$:

(28) $$F_{\mathrm{PGMF}}=\mathrm{Conv}1\left(F_{F_{1},F_{2}}^{1}+\mathrm{UP}\left(\mathrm{Conv}1\left(F_{F_{1},F_{2}}^{2}+\mathrm{UP}\left(F_{F_{1},F_{2}}^{3}\right)\right)\right)\right)$$

By integrating group-wise multi-scale extraction, intra-group cross-modal interaction, and pyramid progressive fusion, the proposed PGMF module effectively enhances both local details and global contextual representation, providing robust features for subsequent voxel-wise weighted averaging fusion and 3D IDWT reconstruction. To further improve readability, we provide a compact pseudocode summary of the GLFC and PGMF modules in Algorithm 1. This algorithm summarizes the main computational flow corresponding to Equations (8)–(28), allowing readers to understand the proposed modules from an implementation-oriented perspective.

**Algorithm 1**: Summary of GLFC and PGMF Procedures

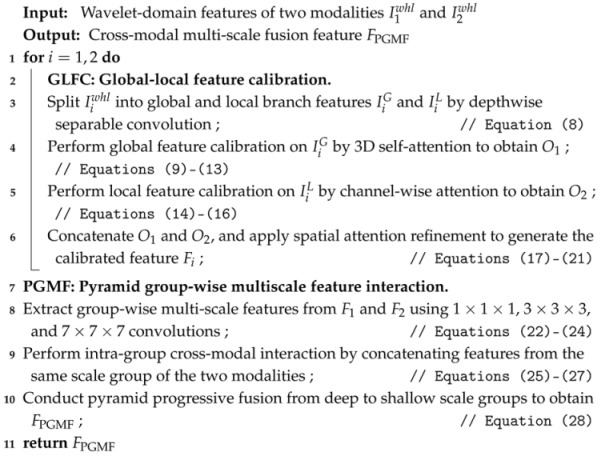



### 2.5. Voxel-Wise Weighted Averaging and 3D Inverse DWT (3D IDWT) Reconstruction Module

In conventional multimodal medical image fusion, non-zero voxels often appear in the background region of the fused image, which are originally zero-intensity areas in the source images. Such inaccurate fusion degrades the quality of the final result and may introduce interference to subsequent clinical diagnosis and analysis. To address this issue, we design a reconstruction framework based on a voxel-wise weighted averaging strategy and 3D inverse discrete wavelet transform (3D IDWT). The input of this module is $F_{\mathrm{PGMF}}$, the final multi-scale cross-modal fusion feature output by the PGMF module, which has already encoded complementary anatomical structure information, fine-grained lesion details and multi-scale contextual dependencies from the two source modalities. Through the proposed framework, we map the high-dimensional fusion feature to adaptive voxel-wise fusion weights, and complete wavelet-domain fusion and image reconstruction while preserving the zero-intensity property of the background region.

The high-dimensional semantic feature $F_{\mathrm{PGMF}}$ cannot be directly used for image reconstruction, as it needs to be mapped to a voxel-wise weight space that matches the spatial size of the original wavelet-domain features. To this end, we design a lightweight weight generation branch based on depthwise separable convolution (DSC) with residual connection, which balances feature representation capability and computational efficiency for 3D volumetric medical data.

For a 3D input feature $X\in R^{C_{in}\times D\times H\times W}$, the 3D DSC operation consists of two sequential steps: 3D depthwise convolution and 3D pointwise convolution, formally defined as:

(29) $$\mathrm{DSC}\left(X\right)={\mathrm{Conv}}_{1\times 1\times 1}\left({\mathrm{DepthConv}}_{3\times 3\times 3}\left(X\right)\right)$$

where ${\mathrm{DepthConv}}_{3\times 3\times 3}$ denotes 3D depthwise convolution with a 3×3×3 kernel, which applies an independent convolution kernel to each input channel to extract spatial features; ${\mathrm{Conv}}_{1\times 1\times 1}$ is 3D pointwise convolution that fuses cross-channel information. Compared with standard 3D convolution, DSC significantly reduces the number of parameters and computational complexity, making it suitable for efficient feature extraction on 3D volumetric medical data.

First, $F_{\mathrm{PGMF}}\in R^{C^{\prime }\times L/2\times H/2\times W/2}$ is fed into the first 3×3×3 DSC layer for initial deep feature extraction, which preserves the spatial resolution while extracting deep fusion features:

(30) $$X_{1}={\mathrm{DSC}}_{1}\left(F_{\mathrm{PGMF}}\right)$$

where ${\mathrm{DSC}}_{1}$ denotes the first DSC layer and $X_{1}$ is the extracted feature with the same spatial size as $F_{\mathrm{PGMF}}$. Then, $X_{1}$ is sent to the second 3×3×3 DSC layer for further feature refinement:

(31) $$X_{2}={\mathrm{DSC}}_{2}\left(X_{1}\right)$$

where ${\mathrm{DSC}}_{2}$ denotes the second DSC layer. To preserve the critical anatomical structure and detail information from the original PGMF output and mitigate the gradient vanishing problem in deep network propagation, we introduce a residual shortcut connection that directly adds the input feature $F_{\mathrm{PGMF}}$ to the refined feature $X_{2}$:

(32) $$X_{\mathrm{res}}=F_{\mathrm{PGMF}}+X_{2}$$

where $X_{\mathrm{res}}$ is the residual-fused feature. This residual fusion mechanism ensures that the global structural prior and local lesion details from the source images are not lost during the convolution transformation, which is of vital importance for the diagnostic value of the final fused image.

Next, the residual-fused feature $X_{\mathrm{res}}$ is processed by a third 3×3×3 DSC layer, which compresses the channel dimension of the feature to one, mapping the high-dimensional fusion feature to a single-channel weight map with exactly the same spatial resolution as the wavelet-domain band features:

(33) $$X_{\mathrm{weight}}={\mathrm{DSC}}_{3}\left(X_{\mathrm{res}}\right)$$

where ${\mathrm{DSC}}_{3}$ denotes the third DSC layer and $X_{\mathrm{weight}}\in R^{1\times L/2\times H/2\times W/2}$ aligns perfectly with the spatial size of the 3D DWT outputs $I_{1}^{whl}$ and $I_{2}^{whl}$. After that, a Sigmoid activation function is applied to normalize the weight map into the range of [0,1], generating the final voxel-wise weight mask *m*:

(34) $$m=\sigma \left(X_{\mathrm{weight}}\right)$$

where σ(·) denotes the Sigmoid activation function. Each element $m_{d,h,w}\in m$ corresponds to a voxel at position (d,h,w) in the 3D volume, and its value represents the adaptive contribution weight of Modality 1 at that voxel, while $1-m_{d,h,w}$ naturally corresponds to the contribution weight of Modality 2. This adaptive weight learning mechanism enables the network to automatically assign higher weights to the modality with richer information at each voxel. Admittedly, more higher weights are assigned to the modality with clearer anatomical structures in the tissue region, while in the zero-intensity background region. The weights of both modalities are adaptively learned to be zero, thus perfectly preserving the zero-value property of the background and completely eliminating non-zero artifacts in the fused image.

With the learned voxel-wise weight mask *m*, we perform weighting fusion in the wavelet domain, which is the core of our reconstruction framework. Different from conventional spatial-domain fusion that easily causes structure blurring and detail loss, wavelet-domain fusion operates on the decoupled structural and detailed features, which can better preserve the complementary information from different modalities. Specifically, we apply the voxel-wise weight mask to the multi-frequency band features of the two modalities directly output by the 3D DWT module. For each voxel position (d,h,w) in the 3D volume, the fusion process is formulated as:

(35) $$I_{f}\left(d,h,w\right)=m\left(d,h,w\right)\cdot I_{1}^{whl}\left(d,h,w\right)+\left(1-m(d,h,w)\right)\cdot I_{2}^{whl}\left(d,h,w\right)$$

The matrix form of the voxel-wise fusion is written as:

(36) $$I_{f}=m⊙I_{1}^{whl}+\left(1-m\right)⊙I_{2}^{whl}$$

where ⊙ denotes element-wise (voxel-wise) multiplication, $I_{1}^{whl}$ and $I_{2}^{whl}$ denote the complete 8-subband wavelet features of Modality 1 and Modality 2, respectively. This fusion strategy ensures that the low-frequency structural information and high-frequency detailed information from both modalities are fused in a decoupled and adaptive manner, avoiding the mutual interference between structural and detailed features during fusion.

Finally, 3D inverse discrete wavelet transform (3D IDWT/3D IWT) is applied to the fused wavelet feature $I_{f}$. As the exact inverse process of the 3D DWT decomposition in the front-end of the network, 3D IDWT sequentially performs 1D inverse DWT along the depth (*L*), height (*H*), and width (*W*) dimensions, reconstructing the eight fused frequency sub-bands back to the original spatial resolution L×H×W of the input source images. The reconstruction process is formally defined as:

(37) $$I_{out}={\mathrm{IDWT}}_{1d}\left({\mathrm{IDWT}}_{1d}\left({\mathrm{IDWT}}_{1d}\left(I_{f}\right)\right)\right)$$

where ${\mathrm{IDWT}}_{1d}\left(\cdot ,dim\right)$ denotes the 1D inverse DWT operation along the specified dimension dim, which is the exact inverse of the 1D DWT used in the 3D DWT module. Benefiting from the perfect reconstruction property of the wavelet transform, this step can restore the fused features to the image space without introducing additional reconstruction error, generating the final fused 3D medical image $I_{out}$ that integrates the complementary anatomical structure information and fine-grained lesion detail information from both modalities.

### 2.6. Loss Function

The design of the loss function should fully consider the characteristics of different modality source images, to ensure that the critical informative content in the source images is well preserved in the fused result. In this work, we adopt a compound loss function consisting of two components: a 3D structural similarity loss $L_{struct}$ to preserve anatomical structural information, and an intensity loss $L_{intens}$ to retain intensity distribution and lesion information. The overall loss function is formulated as:

(38) $$L=\lambda L_{struct}+L_{intens}$$

where λ is a weight parameter that balances the proportion between the structural similarity loss and the intensity loss.

The structural similarity loss $L_{struct}$ is designed based on the Structural Similarity Index Measure (SSIM), which evaluates the similarity between the fused images and the source images in terms of luminance, contrast, and structure. It is formulated as:

(39) $$L_{struct}=\left(1-SSIM(I_{out},I_{1})\right)+\left(1-SSIM(I_{out},I_{2})\right)$$

where $I_{1}$ and $I_{2}$ are the source images of the two modalities, and $I_{out}$ is the final fused image.

The intensity loss $L_{intens}$ is based on the mean squared error (MSE) between the fused image and the source images, which is critical for preserving lesion regions with extremely high or low intensity in medical images. It is defined as:

(40) $$L_{intens}={\left(I_{out}-I_{1}\right)}^{2}+{\left(I_{out}-I_{2}\right)}^{2}$$

The formulation of SSIM can be presented here.

(41) $$\mathrm{SSIM}\left(x,y\right)=\frac{\left(2\mu_{x}\mu_{y}+c_{1}\right)\left(2\sigma_{xy}+c_{2}\right)}{\left(\mu_{x}^{2}+\mu_{y}^{2}+c_{1}\right)\left(\sigma_{x}^{2}+\sigma_{y}^{2}+c_{2}\right)}$$

where $\mu_{x}$ and $\mu_{y}$ denote the mean intensities of images *x* and *y*, $\sigma_{x}^{2}$ and $\sigma_{y}^{2}$ are the corresponding variances, $\sigma_{xy}$ denotes the covariance between *x* and *y*, and $c_{1}$ and $c_{2}$ are small constants (0.0001) used to avoid numerical instability.

## 3. Experiments

### 3.1. Experiment Settings

Our framework is implemented in PyTorch 1.13.1 and trained on an NVIDIA RTX A6000 GPU. To enhance model robustness and prevent overfitting, training samples are randomly cropped into volumetric patches of 64×64×64. We apply a comprehensive data augmentation strategy, including random flipping, scaling, additive white Gaussian noise, and gamma transformation. Due to the large memory footprint of 3D medical volumes, the batch size is set to 1 for both training and testing. We employ the Adam optimizer with an initial learning rate of $1\times 10^{-4}$. A step-decay strategy is adopted, halving the learning rate every 20 epochs over a total of 100 epochs to ensure stable convergence. To rigorously evaluate the performance of the proposed framework, we conduct experiments on two benchmark multimodal medical imaging datasets. BraTS 2020 [29] and Hecktor [30]. The basic visualization found in this experiment are collected from public. These datasets represent distinct clinical challenges in brain and head-and-neck oncology, respectively. The BraTS 2020 dataset focuses on brain glioma using multi-institutional MRI scans, providing four complementary modalities: T1, T2, T1ce, and FLAIR. These modalities offer diverse contrasts crucial for delineating tumor sub-regions, including the necrotic core, peritumoral edema, and enhancing tumor. The BraTS 2020 dataset contains 368 3D multi-contrast MRI volumes. The Hecktor dataset released for the MICCAI 2020 challenge comprises 201 3D CT-PET scans for head and neck squamous cell carcinoma. The structural anatomical details from CT are spatially co-registered with the functional metabolic activity from PET, facilitating integrated metabolic-anatomical analysis. For the BraTS 2020 dataset, we randomly split the 369 volumes into 300 for training and 69 for testing. For the HECKTOR 2020 dataset, we randomly split the 201 paired volumes into 160 for training and 41 for testing. The details can be found in Table 1. All experiments are conducted using five-fold cross-validation to ensure the reliability and generalization of the results.

### 3.2. Comparison Methods and Evaluation Metrics

To rigorously validate the effectiveness of the proposed fusion model, we conduct a comprehensive comparative analysis against eight state-of-the-art (SOTA) deep learning-based multimodal image fusion frameworks. These baselines represent a diverse range of architectural paradigms in the field:

- U2Fusion [10]: A unified unsupervised image fusion network that utilizes deep feature extraction to maintain adaptive information preservation across multiple scenarios.
- SDNet [31]: A squeeze-and-decomposition network designed to decouple source images into salient and discrete components for high-fidelity feature integration.
- IFCNN [32]: A general image fusion framework based on convolutional neural networks that provides a versatile pipeline for multimodal medical data.
- DDcGAN [33]: A dual-discriminator conditional generative adversarial network that leverages adversarial learning to balance the intensity distribution and gradient information between different modalities.
- CSCS [34]: A novel multiscale-decomposition-based fusion method for volumetric medical images, which constructs a cross-scale fusion rule by considering intrascale and interscale consistencies and selects optimal coefficients via neighborhood information utilization.
- LRFNet [35]: A real-time lightweight residual fusion network for multimodal medical image fusion, which employs a three-branch feature extraction framework to preserve brightness and texture information and uses lightweight residual units to greatly improve fusion efficiency.
- LPM-Net [36]: A lightweight pixel-level modeling network for 3D medical image fusion that combines dense CNN and axis-decomposed Mamba for efficient feature extraction.

For a fair comparison, all baseline models are retrained or fine-tuned on the same datasets (BraTS 2020 [29] and Hecktor [30]) using their publicly available source code. For the 2D-based models (e.g., U2Fusion and IFCNN), we employ a slice-by-slice fusion strategy followed by volumetric stacking to accommodate the 3D nature of our test data. CSCS is a traditional optimization-based method following its original setting. The quantitative assessment of fusion performance is conducted using four widely recognized objective metrics, each capturing distinct aspects of information integration. To comprehensively evaluate the performance of multimodal medical image fusion, we adopt four widely used objective metrics, including Localized Mutual Information (LMI), edge information retention metric (QAB/F), Yang’s quality metric (QY), and Visual Information Fidelity for Fusion (VIFF). These metrics evaluate the fused image from different perspectives, including information transfer, edge preservation, luminance consistency, and perceptual fidelity.

Localized Mutual Information (LMI) [37] measures the amount of information transferred from the source images to the fused image within local image regions. Compared with global mutual information, LMI computes information dependency in local neighborhoods and is therefore more sensitive to local anatomical structures and lesion regions. Given two source images $I_{1}$ and $I_{2}$ and the fused image $I_{F}$, LMI evaluates whether the fused image preserves complementary information from both modalities. A larger LMI value indicates that more local information from the source modalities is retained in the fused result. In multimodal medical image fusion, this metric is important because diagnostic regions, such as tumor boundaries or metabolic hotspots, are often localized and spatially heterogeneous.

Edge information retention metric (QAB/F) [38] evaluates how well the edge and gradient information from the source images is preserved in the fused image. It measures the similarity of edge strength and orientation between the source images and the fused image. A higher QAB/F value indicates better preservation of structural boundaries and fine details. This metric is particularly relevant for medical image fusion because anatomical boundaries, lesion margins, and tissue interfaces are critical for clinical interpretation and downstream tasks such as segmentation.

Yang’s quality metric (QY) [39] measures the structural and luminance consistency between the fused image and the source images. It evaluates whether the fused image maintains the brightness distribution and local structural similarity of the input modalities. A higher QY value indicates better visual consistency and fewer artificial distortions. In multimodal medical image fusion, this metric is useful for assessing whether the fusion process introduces unnatural intensity changes or artifacts that may affect clinical readability.

Visual Information Fidelity for Fusion (VIFF) [38] is a perceptual quality metric based on visual information fidelity. It measures how much visual information from the source images is preserved in the fused image from the perspective of the human visual system. A higher VIFF value indicates better perceptual quality and visual fidelity. This metric is suitable for medical image fusion because fused images should not only preserve quantitative information but also remain visually interpretable for clinicians.

### 3.3. Comparison of Model Performance on BraTS2020

**Figure 4:**
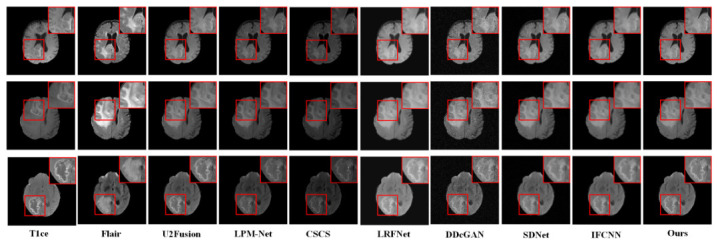
Two-dimensional fusion result comparison on the BraTS2020 dataset.

In addition to subjective visual assessment, comprehensive objective quantitative evaluation is performed to validate the fusion performance of the proposed method on the BraTS2020 dataset. Table 2 summarizes the quantitative results of the proposed model and eight mainstream and state-of-the-art comparative methods (including U2Fusion, SDNet, IFCNN, DDcGAN, CSCS, LRFNet, and LPM-Net) across two typical multimodal pairs, T1/T2 and T1ce/Flair. Four widely recognized metrics, namely LMI, QAB/F, QY, and VIFF, are adopted to comprehensively evaluate the fusion performance from the perspectives of information retention, edge and detail preservation, luminance consistency, and visual information fidelity, with all results averaged over ten test samples to ensure statistical reliability. Furthermore, the visualization result can be found in Figure 4. Compared with other methods, our 3DWaFusion achieves the best balance between structural clarity and lesion detail preservation. The zoomed-in tumor regions show that our method captures fine textures of the enhancing tumor core more accurately, avoiding over-smoothing and preserving the edge contrast critical for clinical diagnosis.

Overall, the proposed method achieves the optimal performance across all four metrics for both T1/T2 and T1ce/Flair modality pairs, comprehensively outperforming all comparative methods. Compared with the widely used classic fusion methods, the proposed method exhibits a consistent and significant performance advantage. These comprehensive improvements demonstrate that the proposed method not only maximizes the retention of complementary information from multimodal source images but also effectively preserves fine edge details, maintains natural luminance consistency, and ensures high visual fidelity of the fused results, which are critical for clinical medical image analysis.

The comprehensive quantitative results on the BraTS2020 dataset consistently validate that the proposed method achieves superior multimodal medical image fusion performance over mainstream and state-of-the-art methods, with excellent generalization ability across different modality pairs, making it well-suited for clinical computer-aided diagnosis tasks that require high-fidelity multimodal image fusion.

### 3.4. Quantitative Comparison of Model Performance on Hecktor

**Figure 5:**
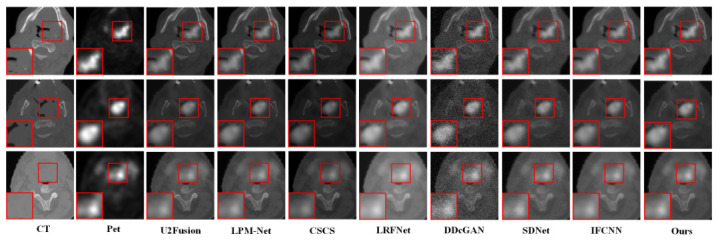
Two-dimensional fusion result comparison on the Hecktor dataset.

In addition to subjective visual assessment, comprehensive objective quantitative evaluation is further conducted to verify the generalization ability of the proposed method on the Hecktor dataset. Table 3 summarizes the quantitative fusion results of the proposed model and eight mainstream and state-of-the-art comparative methods (including U2Fusion, SDNet, IFCNN, DDcGAN, CSCS, LRFNet, and LPM-Net) for the CT/PET multimodal pair. Four widely recognized metrics (LMI, QAB/F, QY, VIFF) are adopted to evaluate the fusion performance from the perspectives of information retention, edge and detail preservation, illumination consistency, and visual information fidelity, with all results averaged over ten test samples to ensure statistical reliability. Furthermore, 2D slice fusion results are demonstrated in Figure 5 for better visualization.

Furthermore, the proposed method exhibits consistent and significant superiority over mainstream fusion methods with different technical routes on the cross-modal CT/PET volumetric fusion task. For U2Fusion and IFCNN, which are widely used 2D-based fusion methods originally designed for 2D image fusion tasks, they have inherent limitations when directly applied to 3D volumetric medical image datasets. These 2D frameworks cannot effectively model the inter-slice spatial context and 3D structural continuity of medical images, leading to insufficient collaborative preservation of fine anatomical details from CT images and functional lesion information from PET images. Quantitatively, the proposed method outperforms U2Fusion by 1.5% in LMI, 4.7% in QAB/F, and 3.5% in VIFF, and surpasses IFCNN by 3.3% in LMI, 5.3% in QAB/F, and 5.5% in VIFF across all metrics, demonstrating the effectiveness of our 3D-aware feature fusion strategy. For the traditional multi-scale decomposition-based CSCS method, the proposed method outperforms it in all evaluation metrics by a notable margin, as the hand-crafted fusion rules of CSCS limit its adaptive feature interaction capability for heterogeneous anatomical and functional information in cross-modal medical images. Compared with LRFNet, a lightweight real-time medical image fusion network, the proposed method achieves better comprehensive performance in all metrics while maintaining competitive inference efficiency, overcoming the limitation of insufficient cross-modal and 3D spatial feature extraction capability in lightweight 2D-oriented models. Even compared with LPM-Net, the state-of-the-art 3D medical image fusion network, the proposed method still achieves better overall performance across all four evaluation dimensions, with particularly prominent advantages in edge detail preservation and cross-modal complementary information fusion, verifying the robustness and generalization ability of the proposed method across different medical imaging modalities and volumetric fusion scenarios.

The comprehensive quantitative results on the Hecktor dataset consistently validate that the proposed method achieves superior multimodal medical image fusion performance on CT/PET cross-modal fusion task, with excellent generalization ability across different imaging modalities and clinical scenarios, making it well-suited for various clinical computer-aided diagnosis tasks that require high-fidelity multimodal image fusion.

### 3.5. Ablation Study

To verify the effectiveness of the proposed Global and Local Feature Calibration (GLFC) module and Pyramid Group-wise Multiscale Feature Interaction (PGMF) module in the fusion network, we conduct two sets of ablation experiments. We use the with/without setting for these two module based on same experimental settings.

#### 3.5.1. Effectiveness of Global and Local Feature Calibration (GLFC) Module

The GLFC module aims to capture rich wavelet-domain information in medical images through dual-path calibration to extract key anatomical and lesion features. As shown in Table 4 and Table 5, compared with the fusion network without the GLFC module, the average performance gains of GLFC on the two datasets in terms of LMI, QAB/F, QY, and VIFF are 1.4%, 7.3%, 2%, and 1.4%, respectively. This demonstrates that the GLFC module can effectively enhance feature discriminability and consistency, thereby improving the quality of multimodal medical image fusion.

#### 3.5.2. Effectiveness of Pyramid Group-Wise Multiscale Feature Interaction (PGMF) Module

The PGMF module aims to improve feature representation ability through group-wise multi-scale and cross-modal interaction. As shown in Table 6 and Table 7, the average performance gains of the PGMF module on the two datasets in terms of LMI, QAB/F, QY, and VIFF are 2.1%, 4.6%, 1.5%, and 1.4%, respectively. This demonstrates that the PGMF module achieves effective exploitation of cross-modal and cross-scale complementary information.

### 3.6. Parameter Analysis of λ

To investigate the influence of λ, we conduct a parameter sensitivity analysis by testing different values from the candidate set {0.1,0.2,0.5,0.7,0.9}. The quantitative results are reported in Table 8. When λ is too small, the structural similarity loss is insufficiently emphasized, leading to weaker anatomical boundary preservation and lower edge-related metrics. As λ increases, the structural consistency of the fused images is gradually improved. However, when λ is excessively large, the network tends to overemphasize structural similarity, which may suppress modality-specific intensity details and lesion contrast. As a result, the performance no longer improves and may even slightly decrease.

As shown in Table 8, λ=0.7 achieves the best overall performance in terms of LMI, QAB/F, QY, and VIFF. Therefore, we set λ=0.7 in all experiments. This value provides a favorable trade-off between anatomical structure preservation and intensity fidelity, and is fixed across all datasets and modality pairs to ensure a fair and consistent evaluation.

### 3.7. Convergence and Complexity Analysis

**Figure 6:**
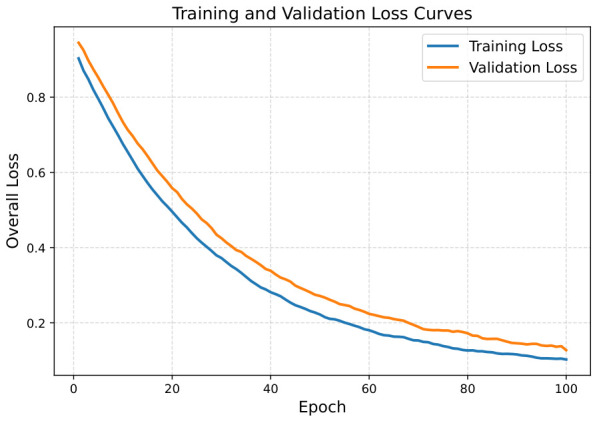
Training-process analysis of the proposed 3DWaFusion framework under BraTS2020 dataset. The curves show the epoch-wise training loss and validation loss. The smooth convergence of the loss curves and the stable increase of the validation metrics demonstrate the effectiveness and stability of the training process.

To further evaluate the quality and stability of the training process, we record the epoch-wise training and validation curves of the proposed 3DWaFusion framework. Specifically, the overall loss function are monitored during training and validation. The corresponding curves are shown in Figure 6.

As can be observed, the training loss decreases smoothly as the number of epochs increases, indicating that the proposed framework can be optimized in a stable manner. Meanwhile, the validation loss follows a similar decreasing trend and gradually reaches a stable plateau, which suggests that the model achieves good convergence without obvious overfitting.

To evaluate the computational efficiency and resource consumption of the proposed 3DWaFusion, we compare it with several state-of-the-art deep learning-based multimodal medical image fusion methods in terms of model parameters (Params), computational complexity (FLOPs), inference speed. The results are summarized in Table 9.

As shown in Table 9, the proposed 3DWaFusion achieves the balanced performance in terms of model parameters, computational complexity, inference speed, and memory consumption. These results demonstrate that this specific design significantly improve the efficiency of our method, making it suitable for clinical applications with limited computing resources.

### 3.8. Segmentation Validation of Fusion Results

**Figure 7:**
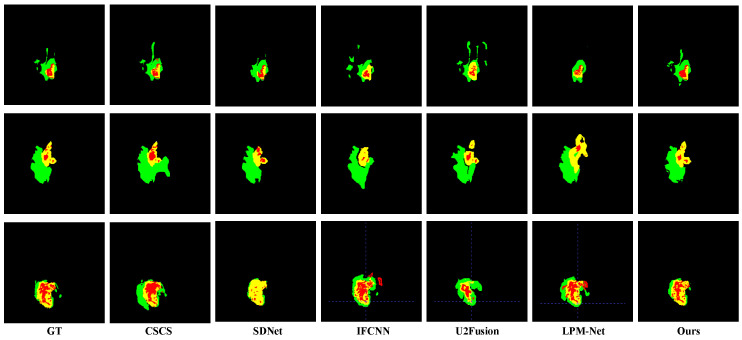
Two-dimensional segmentation result comparison on the BraTS2020 dataset.

To verify the effectiveness of the proposed 3DWaFusion method on downstream clinical tasks, we conduct segmentation validation on the fused images generated by different fusion models. Specifically, we train a unified fixed 3D VNet [40] segmentation model for this evaluation experiment, which is trained and tested on BraTS2020 and Hecktor. This setting ensures a fair comparison, as the only variable is the fusion method, eliminating the impact of different segmentation network architectures. The 2D slices are demonstrated in Figure 7 for better visualization on the BraTS2020 dataset.

In this figure, the first column is the ground truth (GT), and other columns are the segmentation results of the unified VNet model fed with fused images from CSCS, SDNet, IFCNN, U2Fusion, LPM-Net and our proposed 3DWaFusion, respectively. Different rows represent different test samples. Through overall comparison, we can find that the overall structure segmented by our method is closer to GT than all other fusion methods, especially in the Peritumoral edema (ED) and GD-enhancing tumor (ET) regions. Although there is still a slight gap with GT in segmenting some small-area, the segmentation performance of our method is still the best among all methods. These results demonstrate the effectiveness of our proposed fusion method in multimodal brain tumor segmentation tasks.

In addition to subjective visual comparison, we also conduct quantitative analysis in Table 10 show the Dice coefficient and Hausdorff Distance (HD, unit: mm) of the segmentation results from the VNet model, using fused images from different methods on the BraTS2020 datasets. Compared with the other five fusion methods, our proposed 3DWaFusion achieves the best average Dice of 0.7930, which is 0.59% higher than the second-best result of 0.7883 from U-Fusion under GD-enhancing tumor (ET is Label 3), the peritumoral edema (ED is Label 2), and the necrotic and non-enhancing tumor core (NCR/NET is Label 1). This indicates that our fusion method has advantages in the accuracy of segmentation tasks and detail preservation.

To further evaluate the stability of the downstream segmentation from fusion methods, we conduct an additional variance analysis based on five-fold independently trained fusion models. It should be noted that the segmentation network is fixed in this experiment. Specifically, a pre-trained 3D VNet is used as an unchanged downstream evaluator, and no re-training or fine-tuning of the segmentation network is performed for different fusion methods. Therefore, the segmentation results mainly reflect the quality and stability of the fused images rather than the randomness introduced by segmentation model training.

**Figure 8:**
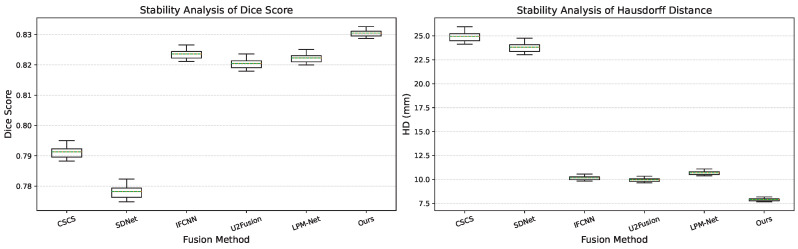
Stability analysis of downstream segmentation performance using a fixed pre-trained 3D VNet. The box plots show the performance variance over five-fold cross validation. The proposed 3DWaFusion achieves consistently higher Dice scores and lower HD values with smaller variance, demonstrating stable downstream segmentation improvement.

For each fusion method, we generate fused images using models trained under five-fold cross validation. The same test cases for segmentation and evaluation protocol are used for all fusion methods. We report the mean and standard deviation of Dice and Hausdorff Distance (HD) across multiple runs. As shown in Figure 8, the proposed 3DWaFusion achieves consistently higher Dice scores and lower HD values than competing methods, while maintaining relatively small performance variance. These results indicate that the segmentation improvement brought by 3DWaFusion is stable and does not rely on a specific random initialization or data split of the fusion model.

## 4. Conclusions

In this work, we proposed a novel 3D Multi-scale Wavelet Convolutional Neural Network (3DWaFusion) for end-to-end 3D multimodal medical image fusion. By leveraging 3D discrete wavelet transform (3D DWT) for multi-frequency decomposition, global and local feature calibration (GLFC) for feature enhancement, pyramid group-wise multi-scale feature interaction (PGMF) for cross-modal information mining, and voxel-wise weighted averaging with 3D inverse discrete wavelet transform (3D IDWT) for artifact-free reconstruction, the proposed framework effectively suppresses spatial redundancy, enhances complementary anatomical and lesion features, and eliminates background artifacts. Extensive experiments on the BraTS2020 and Hecktor datasets demonstrate that 3DWaFusion outperforms state-of-the-art fusion methods in both subjective visual quality and objective quantitative metrics. Furthermore, downstream segmentation validation confirms that the fused images generated by our method significantly improve tumor segmentation accuracy, highlighting the clinical value of the proposed approach. Future work will focus on developing adaptive wavelet decomposition strategies, extending the framework to multiple inputs multimodal fusion tasks, and optimizing the model for real-time deployment on embedded clinical devices to facilitate its translation into clinical practice.

## Figures and Tables

**Table 1:** Summary of the datasets and data partitions used in our experiments.

Item	BraTS2020	HECKTOR2020
Modalities	T1, T2, T1ce, FLAIR	CT, PET
Fusion Task	T1/T2 and T1ce/FLAIR fusion	CT/PET fusion
Total Volumes	369 3D MRI volumes	201 paired 3D volumes
Data Split	300 train/69 test	160 train/41 test

**Table 2:** Quantitative fusion performance comparison on the BraTS2020 dataset. The best results are highlighted in bold.

Method	BraTS2020-Test (T1/T2)				BraTS2020-Test (T1ce/Flair)			
	LMI	QAB/F	QY	VIFF	LMI	QAB/F	QY	VIFF
U2Fusion	0.8046	0.4689	0.9117	0.5588	0.7594	0.4725	0.9260	0.7123
SDNet	0.8123	0.4712	0.9277	0.5621	0.7611	0.4783	0.9216	0.6612
IFCNN	0.8004	0.4356	0.9206	0.5617	0.7703	0.4634	0.9234	0.5236
DDcGAN	0.7924	0.3923	0.8897	0.4365	0.7272	0.4019	0.8942	0.5539
CSCS	0.8012	0.4586	0.9185	0.5479	0.7538	0.4612	0.9207	0.6814
LRFNet	0.8075	0.4814	0.9269	0.5923	0.7589	0.4765	0.9284	0.7016
LPM-Net	0.8136	0.4982	0.9341	0.6015	0.7603	0.4892	0.9346	0.7108
**Ours**	**0.8183**	**0.5274**	**0.9437**	**0.6225**	**0.7625**	**0.5071**	**0.9391**	**0.7645**

**Table 3:** Quantitative fusion performance comparison on the Hecktor dataset. The best results are highlighted in bold.

Method	Hecktor-Test (CT/PET)			
	LMI	QAB/F	QY	VIFF
U2Fusion	0.7825	0.4947	0.9097	0.6225
SDNet	0.7754	0.5000	0.8926	0.6198
IFCNN	0.7685	0.4918	0.8917	0.6107
DDcGAN	0.7532	0.3025	0.8776	0.5567
CSCS	0.7862	0.4925	0.9018	0.6204
LRFNet	0.7911	0.5057	0.9056	0.6347
LPM-Net	0.7926	0.5102	0.9089	0.6385
**Ours**	**0.7942**	**0.5181**	**0.9111**	**0.6442**

**Table 4:** Performance comparison of ablation study for the effectiveness of GLFC module on BraTS dataset.

Model	BraTS2020-Test (T1/T2)				BraTS2020-Test (T1ce/Flair)			
	LMI	QAB/F	QY	VIFF	LMI	QAB/F	QY	VIFF
Without GLFC	0.8066	0.4839	0.9237	0.6084	0.7578	0.4825	0.9117	0.7439
Ours	0.8183	0.5274	0.9437	0.6225	0.7625	0.5071	0.9391	0.7645

**Table 5:** Performance comparison of ablation study for the effectiveness of GLFC module on Hecktor dataset.

Model	Hecktor-Test (CT/PET)			
	LMI	QAB/F	QY	VIFF
Without GLFC	0.7772	0.4811	0.9028	0.6369
Ours	0.7942	0.5181	0.9111	0.6442

**Table 6:** Performance comparison of ablation study for the effectiveness of PGMF module on BraTS dataset.

Model	BraTS2020-Test (T1/T2)				BraTS2020-Test (T1ce/Flair)			
	LMI	QAB/F	QY	VIFF	LMI	QAB/F	QY	VIFF
Without PGMF	0.7828	0.4920	0.9217	0.6025	0.7601	0.4833	0.9311	0.7526
Ours	0.8183	0.5274	0.9437	0.6225	0.7625	0.5071	0.9391	0.7645

**Table 7:** Performance comparison of ablation study for the effectiveness of PGMF module on Hecktor dataset.

Model	Hecktor-Test (CT/PET)			
	LMI	QAB/F	QY	VIFF
Without PGMF	0.7832	0.5091	0.8997	0.6336
Ours	0.7942	0.5181	0.9111	0.6442

**Table 8:** Parameter analysis of λ on the BraTS2020 dataset. The best results are highlighted in bold.

λ	LMI ↑	QAB/F ↑	QY ↑	VIFF ↑
0.1	0.8057	0.4936	0.9294	0.6038
0.2	0.8112	0.5089	0.9356	0.6127
0.5	0.8156	0.5213	0.9408	0.6194
0.7	**0.8183**	**0.5274**	**0.9437**	**0.6225**
0.9	0.8169	0.5231	0.9419	0.6202

**Table 9:** Comparison of model parameters, computational complexity, and inference speed among different DL-based fusion methods on the BraTS2020 dataset.

Method	Params (M)	FLOPs (G)	Runtime (s/vol)
U2Fusion	0.093	412.50	0.0042
SDNet	0.187	672.30	0.0051
IFCNN	0.214	1480.82	0.0025
DDcGAN	0.352	925.40	0.0063
CSCS	0.128	543.20	0.0048
LRFNet	0.031	181.62	0.3333
LPM-Net	0.022	37.65	0.0111
Ours (3DWaFusion)	0.019	8.14	0.0029

**Table 10:** Quantitative segmentation performance comparison of different fusion methods on the BraTS2020 dataset (all results are generated by the 3D VNet model).

Fusion Method	Dice				HD (mm)			
	Label 1	Label 2	Label 3	Avg	Label 1	Label 2	Label 3	Avg
CSCS	0.7102	0.8572	0.8066	0.7913	49.8412	12.0435	12.9817	24.9555
SDNet	0.6914	0.8614	0.7820	0.7782	42.9703	13.3631	15.1033	23.8122
IFCNN	0.7855	0.8950	0.7902	0.8236	12.7524	5.4322	12.2836	10.1561
U2Fusion	0.7775	0.8966	0.7875	0.8205	11.8802	6.2154	11.7862	9.9606
LPM-Net	0.7629	0.9024	0.8016	0.8223	13.7820	6.0287	12.2789	10.6965
Ours (3DWaFusion)	0.7793	0.9036	0.8086	0.8305	8.1066	6.8221	8.7332	7.8873

## Data Availability

The BraTS 2020 dataset used in this study is publicly available from the Center for Biomedical Image Computing and Analytics (CBICA) at the University of Pennsylvania, via the official challenge website: https://www.med.upenn.edu/cbica/brats2020/ (accessed on 25 December 2025). The HECKTOR 2020 dataset is publicly available from the official platform of the MICCAI 2020 HECKTOR Challenge on Grand Challenge: https://hecktor.grand-challenge.org/ (accessed on 25 December 2025). All datasets are accessible under the terms of their original open-access data usage licenses.
